# Parameter Estimation of SAR Signal Based on SVD for the Nyquist Folding Receiver

**DOI:** 10.3390/s18061768

**Published:** 2018-06-01

**Authors:** Tao Li, Qian Zhu, Zengping Chen

**Affiliations:** College of Electronic Science, National University of Defense Technology, Changsha 410073, China; litao_nudt@163.com (T.L.); atrchen@sina.com (Z.C.)

**Keywords:** Nyquist Folding Receiver, LFM signal, parameter estimation, singular value decomposition, golden section method

## Abstract

The Nyquist Folding Receiver (NYFR) is a novel ultra-wideband (UWB) receiver structure that can realize wideband signal monitoring with fewer components. The NYFR induces a Nyquist zone (NZ)-dependent sinusoidal frequency modulation (SFM) by a modulated local oscillator (LOS), and the intercepted linear frequency modulated (LFM) synthetic aperture radar (SAR) signal will be converted into an LFM/SFM hybrid modulated signal. In this paper, a parameter estimation algorithm is proposed for the complicated NYFR output signal. According to the NYFR prior information, a chirp singular value ratio (CSVR) spectrum method based on singular value decomposition (SVD) is proposed to estimate the chirp rate directly before estimating the NZ index. Then, a fast search algorithm based on golden section method for the CSVR spectrum is analyzed, which can obviously reduce the computational complexity. The simulation shows that the presented algorithm can accurately estimate the parameters of the LFM/SFM hybrid modulated output signal by the NYFR.

## 1. Introduction

Ultra-wideband (UWB) receivers [1] need to achieve a high-probability reception of input signals over an extremely wide bandwidth. In addition to using ultrafast sampling methods or devices, UWB can be implemented with sub-Nyquist sampling techniques, such as modified Gegenbauer system [1,2], Xampling [3,4], and analog-to-information convertors (AIC) [5,6,7,8]. Among them, the AIC architecture based on compressive sensing (CS) theory allows sampling of only useful information at lower sampling rates without large hardware scale and has proven to be an effective sampling method for sparse signals. The Nyquist Folding Receiver (NYFR) has recently been proposed as a special type of AIC that performs radio frequency (RF) spectrum compression via a periodic non-uniform local oscillator (LOS) and induces a Nyquist zone (NZ)-dependent modulation on the received signal. Unlike the Gegenbauer polynomial method [1,2] or most other CS schemes [7,8,9,10] that require full signal reconstruction, the NYFR substantially preserves signal structure, and the information reconstruction can be relatively simple using conventional signal analysis methods without sparse recovery.

As the most mature signal of synthetic aperture radar (SAR), linear frequency modulated (LFM) signal has the advantage of good concealment, wide bandwidth, low peak power, and strong anti-interference, which makes it widely used in a variety of radar systems [11]. Parameter estimation of LFM signal has been a typical issue in the field of radar and reconnaissance. Conventional processing algorithms can be introduced to the NYFR to deal with intercepted SAR signals and avoid computationally expensive reconstruction. However, existing research on the NYFR have mainly focused on CS methods [12,13,14], and algorithms of signal parameter estimation for the NYFR output signal still require further research due to its special signal type. In conventional NYFR utilizing sinusoidal frequency modulation (SFM) LOS, the intercepted LFM signal will be converted into LFM/SFM hybrid modulated signal. This brings difficulties for back-end data processing.

In Reference [15], analytic wavelet transform was used for NYFR information recovery from the hybrid modulated signal. However, the performance of this algorithm needs further improvement. In Reference [16], a spectrum peak method was proposed for the NYFR, that is, to construct a multi-channel structure and estimate the NZ index by comparing the spectrum peak value of each channel. However, the performance of this algorithm will deteriorate along with the increase of signal bandwidth. In Reference [17], the NYFR output signal was processed based on a complicated time-frequency diagram method. This method allows us to calculate some instantaneous frequencies of the LFM/SFM hybrid modulated signal to get the upper and lower boundary lines of the time-frequency curve. The chirp rate and NZ index can then be estimated by the slope and the NZ-dependent SFM bandwidth. This time-frequency curve algorithm is intuitive but requires a higher signal-to-noise ratio (SNR) as it does not make full use of all the sampled data. Based on the periodicity of SFM, the authors of Reference [18] looked at the use of autocorrelation to process LFM/SFM hybrid modulated signal. The study found that if the interval is exactly the SFM period, a sinusoidal signal with a chirp rate-dependent carrier frequency can be obtained by autocorrelation. Therefore, we can first estimate the chirp rate like fast dechirp algorithm [19], then convert the signal into SFM signal, and lastly estimate the NZ by the spectrum peak method. However, the autocorrelation method is limited to the signal type and requires a higher SNR.

In addition to these methods, to avoid the complicated processing of SFM signal, the authors of Reference [20] adopted periodic LFM signal as the modulated LOS, and the NZ index was estimated via the chirp rate of the NYFR output signal. The method is easy to implement due to the maturity of LFM modulation technology and provides a new design for the NYFR. However, for LFM signal input, the chirp rate of NYFR output signal is derived from the input signal itself and the LFM LOS. Consequently, conventional methods are not applicable for this case, and no better algorithm has been reported. In Reference [21], an improved dual-channel NYFR architecture was proposed to reduce the difficulty of NYFR output signal processing. However, this scheme introduced an auxiliary channel to the NYFR prototype structure, which doubled the hardware size. Thus, it violated the original intention of NYFR to monitor more bandwidth with less hardware.

In this paper, a parameter estimation algorithm based on singular value decomposition (SVD) [22] is proposed for LFM signals intercepted by the NYFR, which makes use of the LOS periodicity to estimate the chirp rate before estimating the NZ index. To reduce the computational complexity, the usability of golden section method and the reasonable step of its rough search are analyzed. Simulation results verify the efficiency of the proposed algorithm for the hybrid modulated signal in the NYFR. This algorithm is also suitable for the NYFR with periodic LFM LOS.

## 2. NYFR Architecture and Intercepted LFM Signal

**Figure 1:**
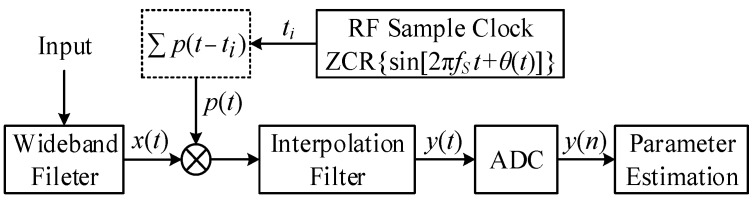
Nyquist Folding Receiver (NYFR) architecture.

The NYFR architecture [6] is shown in Figure 1. The input analog signal x(t) is first filtered by an UWB preselect filter, and then mixed by a non-uniform radio frequency (RF) LOS  p(t), which is controlled by zero-crossing rising (ZCR) voltage time of a RF sample clock. Then, the mixer output signal x(t) is filtered by an interpolation low-pass filter (LPF) with pass band $\left(-f_{s}/2,f_{s}/2\right)$, and we would obtain y(t) as the output of the NYFR. The signal y(t) contains the LOS modulation information that can be measured to determine the original RF band. Next, y(t) is sampled by an analog-to-digital convertor (ADC) to get the discrete NYFR output, and the sampling rate $f_{s}$ is equal to the LOS carrier frequency. Finally, information of the original signal x(t) is recovered by the corresponding parameter estimation method.

For ease of deduction, all the signals are expressed in the form of complex number. We define $\left(-f_{s}/2,f_{s}/2\right)$ as the 0-th NZ, hence, $\left(lf_{s}-f_{s}/2,lf_{s}+f_{s}/2\right),l\in \left\{1,\ldots ,L\right\}$ is the l-th NZ.

In Figure 1, θ(t) represents a narrow-band LOS modulation. According to [16], if SFM is selected as the modulation, the non-uniform LOS p(t) can be normalized and rewritten as:

(1) $$p\left(t\right)=\overset{L-1}{\underset{l=0}{\sum }}\exp\left\{jl\left[2\pi f_{s}t+m_{f}\sin\left(2\pi f_{\sin}t\right)+\varphi_{\mathrm{LOS}}\right]\right\}$$

where $m_{f}$ is the modulation coefficient, $f_{\sin}$ is the modulation frequency, and $\varphi_{\mathrm{LOS}}$ is the LOS initial phase. In essence, p(t) consists of a set of SFM signals located at the center of their respective NZ. Additionally, if periodic LFM is selected as the modulation, the $m_{f}\sin\left(2\pi f_{\sin}t\right)$ in (1) should be replaced by a LFM signal type.

Here, we denote LFM signal as the NYFR input, and it can be expressed as:

(2) $$x\left(t\right)=A\exp\left\{j\left[2\pi \left(f_{0}t+0.5\mu_{0}t^{2}\right)+\varphi_{0}\right]\right\}+w\left(t\right)$$

where  A, $f_{0}$, $\mu_{0}$ and $\varphi_{0}$ are the amplitude, start frequency, chirp rate and initial phase, respectively, and w(t) is the additive white Gaussian noise distributed in the monitoring frequency band. For simplification, it is assumed that the frequency range of the LFM signal does not cross the NZ junctions, i.e., $lf_{s}$.

After mixing, low-pass filtering, and sampling, the discrete expression of NYFR output [16] can be given by:

(3) $$y\left(nT_{s}\right)=A\exp\left[j2\pi \left(f_{0}-l_{\mathrm{NZ}}f_{s}\right)nT_{s}+j\pi \mu_{0}{\left(nT_{s}\right)}^{2}+j\varphi_{0}-jl_{\mathrm{NZ}}m_{f}\sin\left(2\pi f_{\sin}nT_{s}\right)-jl_{\mathrm{NZ}}\varphi_{\mathrm{LOS}}\right]+w\left(nT_{s}\right)$$

where $l_{\mathrm{NZ}}$ is the NZ index indicating the original carrier frequency of the input signal, n=0, 1,…, N−1,$\;T_{s}=1/f_{s}$, and $l_{\mathrm{NZ}}=\mathrm{round}\left[\left(f_{0}+\mu_{0}t\right)/f_{s}\right]$. $w\left(nT_{s}\right)$ is the additive noise that is modulated according to the original NZ position, and its power spectrum is folded into $\left(-f_{s}/2,f_{s}/2\right)$. $y\left(nT_{s}\right)$ is a LFM/SFM hybrid modulated signal to be processed for parameter estimation.

**Figure 2:**
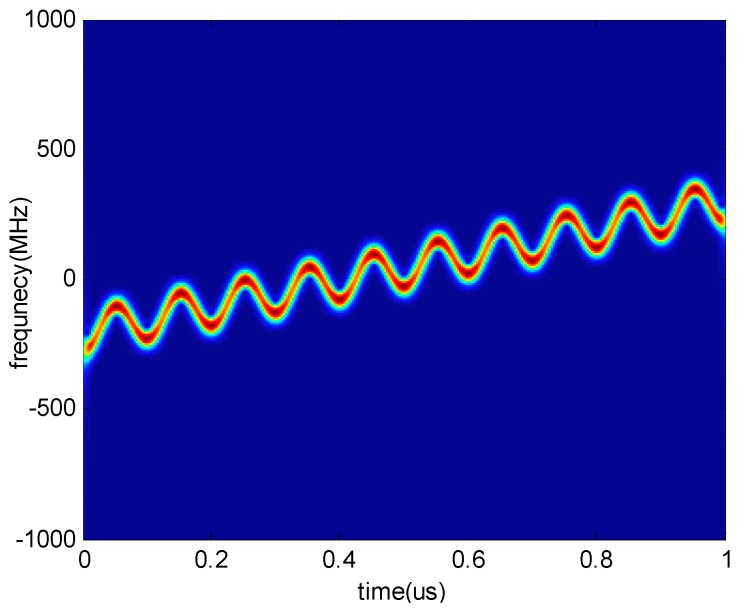
Time-frequency diagram of a sampled linear frequency modulated (LFM) signal.

Figure 2 shows the time-frequency diagram of an LFM signal sampled by the NYFR. The time-frequency curve of the input LFM signal is superimposed on the sinusoidal frequency modulation of the LOS. The modulation parameters of the SFM part correspond to the values of $l_{\mathrm{NZ}}$, which demonstrates the frequency band where the input signal is located. The main parameters estimated in this paper are the chirp rate and the NZ index, which correspond to the slope of the time-frequency curve and bandwidth of the SFM in Figure 2.

## 3. Parameter Estimation Based on SVD

Intercepted by the NYFR with SFM LOS, the LFM signal is converted into LFM/SFM hybrid modulated signal. For the SFM part, only the NZ modulation index $l_{\mathrm{NZ}}$ is unknown. Unlike the existing algorithms [16,18] that treat the LFM/SFM hybrid modulated signal as a simple modulation signal and regard the other modulation as a redundant part, we make full use of the its modulation characteristics, and estimate chirp rate directly using the LOS periodic information.

The LOS modulation period can be calculated as $1/f_{s}$ and the number of points in one LOS modulation period is $N_{\sin}=f_{s}/f_{\sin}$. In addition, $f_{\sin}$ and $f_{s}$ are the prior parameters for the NYFR structure. Thus, we can set $N_{\sin}=f_{s}/f_{\sin}\in {\mathbb{Z}}_{+}$ and $M_{c}=⎣N/N_{\sin}⎦$, where ⎣·⎦ means the floor operation and $M_{c}\in {\mathbb{Z}}_{+}$. The above setting implies that the number of signal points we use in this section is $M_{c}N_{\sin}$, and if the input data length $N>M_{c}N_{\sin}$, we can select $M_{c}N_{\sin}$ points and omit the remaining points. 

For simplification, we omit the noise part temporarily, and abbreviate $nT_{s}$ as n. According to the LOS periodic characteristic, we first observe the relationship between two adjacent LOS periods.

(4) $$\frac{y\left[\left(p+1\right)N_{\sin}+n\right]}{y\left(pN_{\sin}+n\right)}=A\exp\left\{j2\pi \left(f_{0}-l_{\mathrm{NZ}}f_{s}\right)N_{\sin}T_{s}+j\pi \mu_{0}T_{s}{}^{2}N_{\sin}\left[\left(2p+1\right)N_{\sin}+2n\right]\right\}$$

It can be observed that when Equation (4) has no LFM modulation part, the quotient of the elements whose interval is a LOS period will be a constant.

Thus, we can dechirp the signal y(n) in Equation (3) by multiplying an auxiliary LFM signal $s_{\mu }\left(n\right)=\exp\left[-j\pi \mu {\left(nT_{s}\right)}^{2}\right]$ , where *μ* is an argument. The result can be reshape to a $M_{c}\times N_{\sin}$ matrix.

(5) $$Y_{\mu }\left(n\right)=y\left(n\right)*s_{\mu }\left(n\right)$$

(6) $$Y\left(\mu \right)=\left[\begin{array}{c} \begin{array}{ccc} Y_{\mu }\left(1\right) & \cdots & Y_{\mu }\left(N_{\sin}\right)\\ Y_{\mu }\left(N_{c}+1\right) & \cdots & Y_{\mu }\left(2N_{\sin}\right)\\ \vdots & \ddots & \vdots \\ Y_{\mu }\left[\left(M_{c}-1\right)N_{c}+1\right] & \cdots & Y_{\mu }\left(M_{c}N_{\sin}\right) \end{array} \end{array}\right]$$

The singular value decomposition (SVD) of Y(μ) can be computed as $U\Sigma V^{H}$ [21], where Σ is an $M_{c}\times N_{\sin}$ diagonal matrix, and we call it the singular values matrix. The singular values are $\lambda_{1},\lambda_{2},\;\cdots ,\lambda_{M_{c}}$, and $\lambda_{1}\geq \lambda_{2}\geq \cdots \geq \lambda_{M_{c}}$. Considering a noise-free situation, if $\mu =\mu_{0}$, Y(μ) will become an SFM signal matrix whose row elements are proportionate to the data in one LOS modulation period, therefore the first singular value $\lambda_{1}$ will reach its maximum, and the rest singular values will be zeros. If $\mu \neq \mu_{0}$, the periodic characteristic of the LOS in each row of Y(μ) will be weakened by the LFM modulation, and consequently, the other singular values of Y(μ) will be non-zero.

Based on the characteristic above, we define the chirp singular value ratio (CSVR) spectrum [23] as:

(7) $$p\left(\mu \right)=\frac{\lambda_{1}^{2}}{\lambda_{1}^{2}+\lambda_{2}^{2}+\ldots +\lambda_{M_{c}}^{2}}$$

Meanwhile, we can search the peak of CSVR spectrum in Equation (7) whose argument is the chirp rate and the estimated chirp rate is:

(8) $${\hat{\mu }}_{0}=\underset{\mu }{\arg}\{\max[p\left(\mu \right)]\}$$

**Figure 3:**
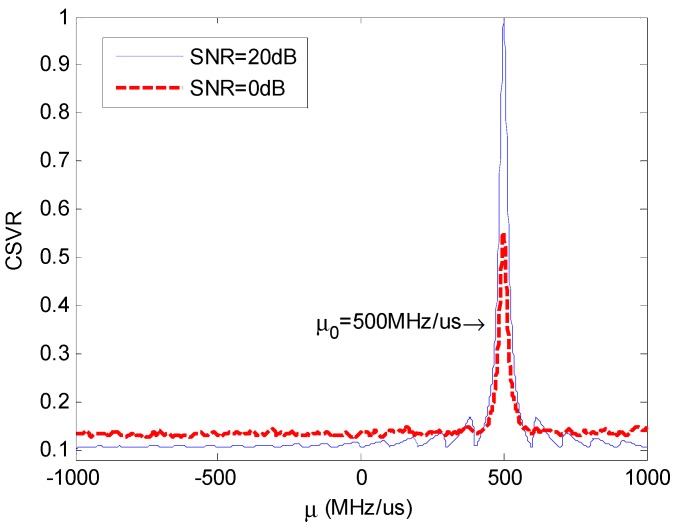
Chirp singular value ratio (CSVR) spectrum under different signal-to-noise ratio (SNR).

Figure 3 shows the relationship between the CSVR spectrum and μ under different SNR.

**Figure 4:**
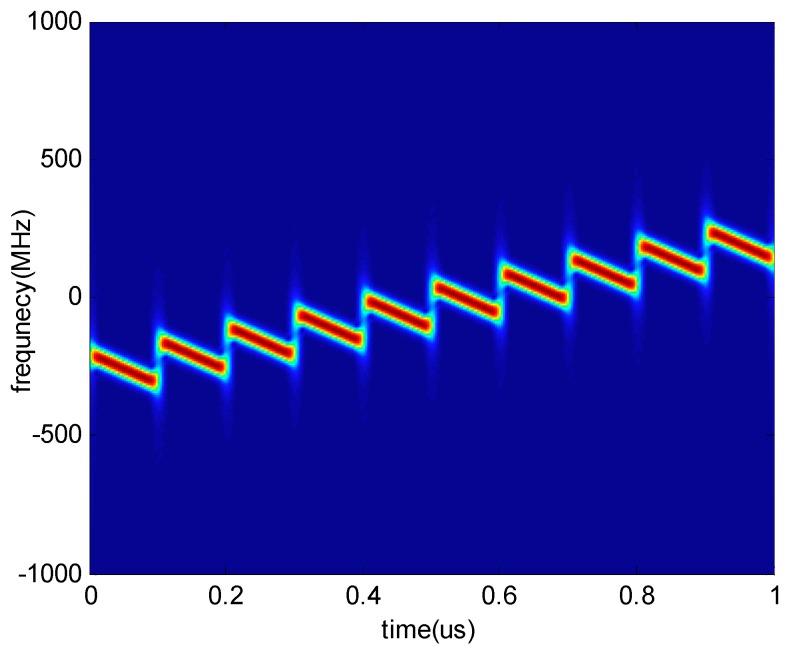
Time-frequency diagram of a sampled LFM signal using periodic LFM local oscillator (LOS).

**Figure 5:**
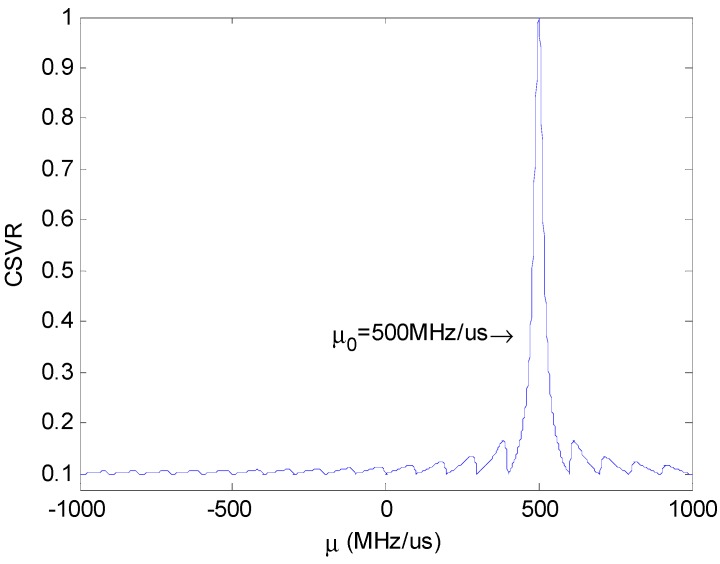
CSVR spectrum using periodic LFM LOS.

It is worth noting that this algorithm is also suitable for the NYFR with periodic LFM LOS. Figure 4 shows the time-frequency curve of an LFM signal intercepted by the NYFR with periodic LFM LOS. The intercepted LFM signal can be processed similar to the above processing, and the CSVR spectrum is shown in Figure 5. The chirp rate can also be estimated by searching the peak of CSVR spectrum so that it can provide a feasible method for processing LFM signals intercepted by the NYFR with periodic LFM LOS.

## 4. Fast Search Algorithm

We can obtain an accurate estimation of $\mu_{0}$ by scanning the CSVR spectrum, and the search interval is not limited by the data length. However, the computational complexity of the proposed method may be larger than the existing methods for two main reasons. First, the SVD processing complexity is high. Second, the total number of SVD processing may be very large, especially if a high precision is required and the search step is small. For the first point, there are some fast SVD methods [24,25] that can reduce the computational complexity of each SVD decomposition and enhance the speed.

To reduce the computational complexity of the complete search of chirp rate, this section discusses the availability of golden section search method for the CSVR spectrum algorithm. Although the dichotomy method has a faster convergence rate than the golden section method under certain circumstances, the dichotomy requires a high symmetry around the peak point to ensure that the iteration is done correctly. Therefore, we choose the golden section method of convergence—which has a lower but more stable convergence rate—to search the exact value of $\mu_{0}$.

An important prerequisite for the golden section method is to find the position near the maximum point in order to avoid iteration to the local extreme point. This can be achieved by a rough search within a reasonable interval. For the rough search, a small interval will lead to a considerable computation, while a large interval will lead to the absence of the main lobe around the true value of $\mu_{0}$, and achieve a wrong estimation. Hence, we resort to the width of the maximum main peak of the CSVR spectrum to determine the maximum available search interval.

According to the Jacobi–Anger expansion, we have:

(9) $$\exp[jl_{\mathrm{NZ}}m_{f}\sin\left(2\pi f_{\sin}nT_{s}\right)]=\overset{\infty }{\underset{m=-\infty }{\sum }}J_{m}\left(l_{\mathrm{NZ}}m_{f}\right)\exp\left(j2\pi mf_{\sin}nT_{s}\right)$$

where $J_{m}\left(\cdot \right)$ is the m-th Bessel function.

According to Equation (9), the SFM part of y(n) in Equation (3) consists of a series of frequency components with a fundamental frequency $f_{\sin}$, so $f_{\sin}$ is the key parameter that determines the periodicity of CSVR spectrum. Here we directly give the expression of the periodic fluctuation interval of CSVR spectrum as Equation (10), and verify it by simulation.

(10) $$\Delta \mu =f_{\sin}^{2}$$

**Figure 6:**
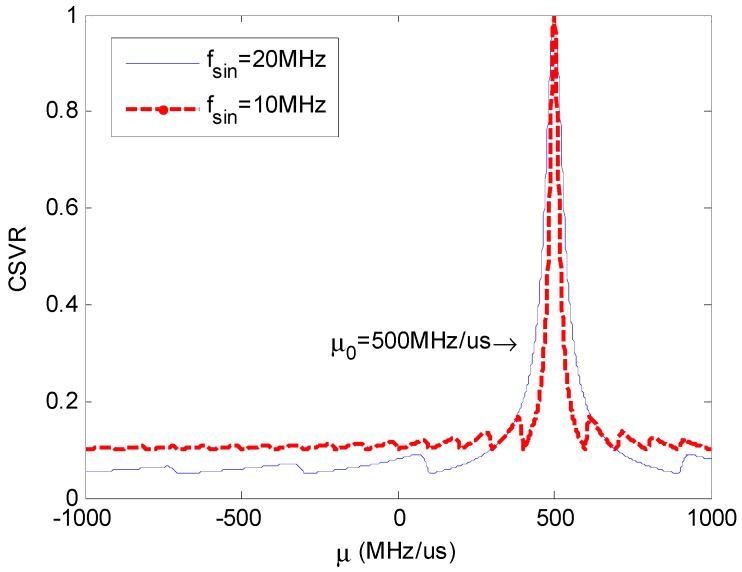
CSVR spectrum using different $f_{\sin}$.

Figure 6 shows two CSVR spectrums, corresponding to $f_{\sin}=10\;\mathrm{MHz}$ and $f_{\sin}=20\;\mathrm{MHz}$ respectively. It can be seen that the periodic fluctuation intervals of CSVR spectrum are 100 MHz/μs and 400 MHz/μs respectively, conforming to Equation (10).

The main lobe width of the SVR spectrum in Figure 6 is $2\Delta \mu =2f_{\sin}^{2}$. In order to improve the accuracy of CSVR spectrum peak search under low SNR, we set the search interval of rough search as 1/4 of the main lobe width, i.e., $f_{\sin}^{2}/2$. Thus, by a rough search of μ in the possible range of chirp rate, we can ensure that the maximum of the results is located within the main lobe of the CSVR-μ curve.

**Figure 7:**
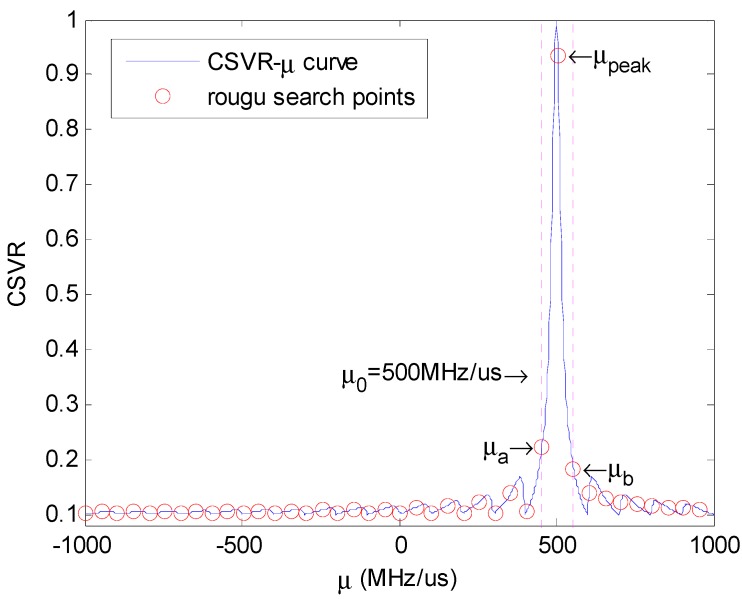
Rough search demonstration.

As Figure 7 shows, the peak point after rough search is $\mu_{\mathrm{peak}}$. Additionally, we can ensure the maximum of CSVR spectrum is located between the left and the right of the $\mu_{\mathrm{peak}}$ point, that is the area between two upright purple lines.

We choose neighboring positions of the peak point of rough search—namely $\mu_{a}$ and $\mu_{b}$—as the initial boundary of the golden section search. Within the initial interval $\left(\mu_{a},\mu_{b}\right)$, the CSVR-μ curve has only one local extreme point, which is also the global maximum point. After setting the precision requirements, the accurate estimation of chirp rate can be finally obtained through iterative search.

It is assumed that the possible range of μ is from −1000 MHz/μs to 1000 MHz/μs, the required estimated accuracy is $10^{-3\;}\;\mathrm{MHz}/\mu s$, and the modulation frequency is $f_{\sin}=10\;\mathrm{MHz}$. A complete direct search on chirp rate needs calculating a total of $2\;\times \;10^{6}$ CSVR values of chirp rate points. By contrast, this fast search method needs a calculation of only 41 times in the rough search, and about 23 times in the iterative search to reach the same precision. Compared with direct search, the proposed search scheme can reduce the amount of computation by more than 99%.

After estimating the chirp rate, we can dechirp the LFM/SFM hybrid modulated signal in Equation (3) to get a SFM signal, and then the NZ index can be estimated by the conventional spectrum peak method [16].

To sum up, for the LFM signal intercepted by the NYFR, the proposed parameter estimation algorithm can be divided into four steps:

1. Rough search of chirp rate based on CSVR with interval $f_{\sin}^{2}/2$ to get its possible range.
2. Fast iterative search of chirp rate based on the golden section method to get the accurate estimation result.
3. Dechirp the LFM/SFM hybrid modulated signal to get the SFM part.
4. NZ index estimation based on spectrum peak method.

## 5. Numerical Experiments

In this section, simulation experiments were conducted to verify the performance of the proposed method using the following parameters listed in Table 1.

**Figure 8:**
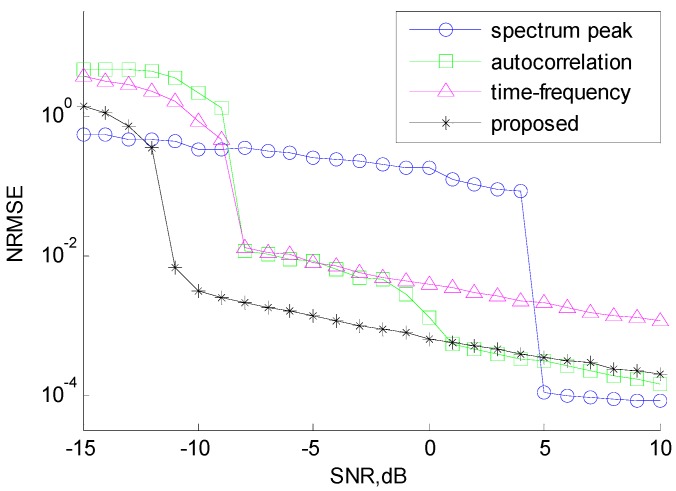
Normalized root mean square errors (NRMSE) of chirp rate estimation.

**Figure 9:**
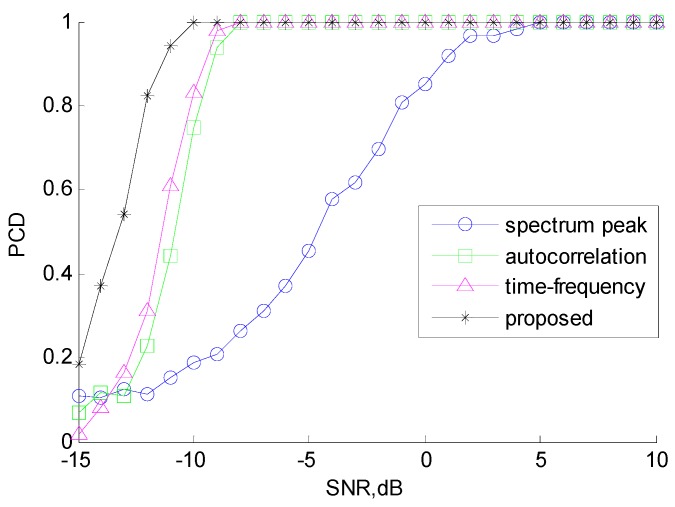
Correct ratio of Nyquist Zone (NZ) index estimation.

We compared the proposed CSVR method with the spectrum peak method [16], the autocorrelation method [18], and the time-frequency curve method described in Section 1. Figure 8 illustrates the normalized root mean square error (NRMSE) of chirp rate estimation. Figure 9 illustrates the performance of NZ index estimation, evaluated by the probability of correct decision (PCD).

It is clear from the figures that the proposed method outperforms the other three methods. With the proposed architecture, the estimation performance of the chirp rate is outstanding and stable when SNR ≥ −10 dB. The correct ratio of NZ index estimation is greater than 90% when SNR ≥ −11 dB and reaches 100% when SNR ≥ −10 dB. This is because the proposed CSVR method effectively utilizes the characteristics of the hybrid modulated signal and can achieve super resolution through iteration. By contrast, the spectrum peak method estimates NZ index disregarding the effect of chirp rate, resulting in a low PCD of NZ index estimation and therefore, the performance of chirp rate estimation is affected by the NZ result. The autocorrelation method results in loss of SNR due to autocorrelation, so the accuracy of chirp rate estimation is poor at low SNR. The time-frequency method only uses the instantaneous frequency values of several moments and does not make full use of all the sampling data, so its performance is also not good.

It should be noted that in Figure 8, after correctly estimating the NZ index when SNR ≥ 5 dB, the spectrum peak method can obtain a higher estimation accuracy of the chirp rate by means of high precision algorithms such as Fractional Fourier Transform (FrFT) [26], which is especially suitable for simple LFM signal. However, it is obvious that the spectral peak method needs more computation, requires much higher SNR threshold, otherwise fails under low SNR. Among the four methods, the proposed SVD-based parameter estimation algorithm has the best robustness to low SNR and the most stable estimation performance.

## 6. Conclusions

On the basis of the LOS periodicity property, a parameter estimation algorithm based on SVD of matrix is proposed for LFM signals intercepted by the NYFR. We make full use of the LOS prior information to estimate the chirp rate before estimating the NZ index. Then, an effective fast search scheme based on the golden section method is put forward to reduce the computational complexity. Simulation results demonstrate the superior performance of the proposed algorithm compared to the existing algorithms for the LFM/SFM hybrid modulated signal in the NYFR. Besides, this algorithm is also suitable for the NYFR with periodic LFM LOS.

## Figures and Tables

**Table 1:** The simulation settings.

Monitoring frequency band	*f*	1~21 GHz
Amount of NZ	L	10
Average sampling rate	$f_{s}$	2 GHz
LOS modulation coefficient	$m_{f}$	2
Sinusoid modulation frequency	$f_{\sin}$	10 MHz
Signal length	T	1 μs
Simulation points	N	2000 points
Signal amplitude	A	1
Chirp rate	μ	500 MHz/μs
Start frequency	$f_{0}$	7.6 GHz
Initial phase	$\varphi_{0}$	0
NZ index	$l_{\mathrm{NZ}}$	4
